# A cortical pathway to sleep: Role of long‐range inhibition

**DOI:** 10.1002/ctm2.70868

**Published:** 2026-10-15

**Authors:** Geoffrey Terral, Renata Batista‐Brito

**Affiliations:** ^1^ Nash Family Department of Neuroscience Icahn School of Medicine at Mount Sinai New York New York USA

1

Identifying the brain regions that govern sleep–wake states has been one of the fundamental questions in neuroscience. For decades, models have emphasised subcortical structures, particularly brainstem, hypothalamus, basal forebrain and thalamus circuits, as central regulators of transitions between sleep and wakefulness.^1^ In this framework, the neocortex has often been considered primarily a downstream recipient of global state signals. Yet, accumulating evidence indicates that cortical circuits themselves can influence sleep, sleep homeostasis and sleep‐associated network dynamics.^2^, ^3^, ^4^ Our recent study expands this emerging view by identifying a sparse population of long‐range cortical inhibitory neurons (INs) that provides a cellular cortical pathway through which the neocortex can contribute to distributed sleep regulation.^5^

The idea that the cortex can regulate sleep–wake states is particularly intriguing because cortical activity has traditionally been viewed primarily as a readout of these states. Indeed, electroencephalographic and local field potential recordings reveal stereotyped patterns of neocortical activity across sleep and wakefulness. Periods of low arousal, such as slow‐wave sleep (SWS) or quiet immobility, are characterised by low‐frequency fluctuations and coordinated cortical spiking and are termed synchronised states. These states alternate with desynchronised states, which occur during periods of high arousal and active behaviour and are characterised by the suppression of low‐frequency cortical fluctuations. These observations raised the possibility that the cortex not only reflects sleep–wake states but also contributes to their regulation through specific neuronal populations. GABAergic INs have been repeatedly suggested as regulators of arousal state‐dependent neocortical activity with fast‐spiking parvalbumin‐expressing INs demonstrated to promote desynchronised states. In contrast, little was known about the function of other INs in the control of synchronised states.

A small and distinct class of neocortical INs, characterised by the co‐expression of somatostatin (*Ss*t) and chondrolectin (*Chodl*), together with neuronal nitric oxide synthase (*Nos1*) and the neurokinin‐1 receptor (*Tacr1*), referred to as Sst‐Chodl cells, has also long been proposed to contribute to sleep regulation.^6^ Whereas most cortical neurons reduce their activity during sleep, previous ex vivo work suggested that Sst‐Chodl cells may be active during sleep recovery,^7^ pointing to a possible relationship between the activity of these cells, synchronised cortical states and behaviour. This raised three central questions: Are Sst‐Chodl cells active during sleep in vivo? Do they causally promote synchronised cortical activity, sleep or both? And do they possess distinctive properties that enable them to perform this function? Despite this longstanding hypothesis, Sst‐Chodl cells have remained difficult to investigate because of the lack of tools to selectively target, monitor and manipulate them.

Using intersectional genetics, it is now possible to selectively target cells that co‐express Cre and Flp recombinases. For example, *Sst‐Flp*, *Chodl‐Cre* mice provide genetic access to Sst‐Chodl cells, enabling their selective monitoring and manipulation, which allowed us addressing the questions abovementioned.^5^

Using in vivo two‐photon calcium imaging in mice, we found that Sst‐Chodl neurons are strongly modulated by behavioural state. Their activity is highest during SWS and quiet wakefulness and lowest during active, high‐arousal states (Figure 1a). Importantly, their activity is not simply a correlate of sleep. Selective optogenetic activation increased low‐frequency cortical activity, enhanced neuronal synchrony and generated coordinated effects within and across distant cortical regions. In contrast, activating another major class of *Sst*‐expressing INs, Martinotti cells (Figure 1b), did not reproduce these effects, emphasising the functional specialisation that exists within broad molecular classes of INs. When Sst‐Chodl neurons were activated broadly across the neocortex using chemogenetic approaches, mice spent more time asleep, entered sleep more rapidly and showed increased intensity of SWS (Figure 1c). The effect was observed not only during the light phase, when mice normally sleep more, but also during their active dark phase. Finally, our anatomical reconstructions confirmed and extended previous descriptions of the distinctive morphology of Sst‐Chodl neurons. Unlike most cortical interneurons, which primarily target local networks, Sst‐Chodl neurons combine dense local arbors with long‐range axons extending over millimeters and innervating multiple regions of the ipsilateral neocortex (Figure 1d), providing an anatomical substrate for influencing distant cortical activity. Together with our functional experiments, these findings suggest that their distinctive long‐range morphology may enable Sst‐Chodl neurons to coordinate synchronised activity across broad cortical territories and thereby promote sleep.^5^

**FIGURE 1:**
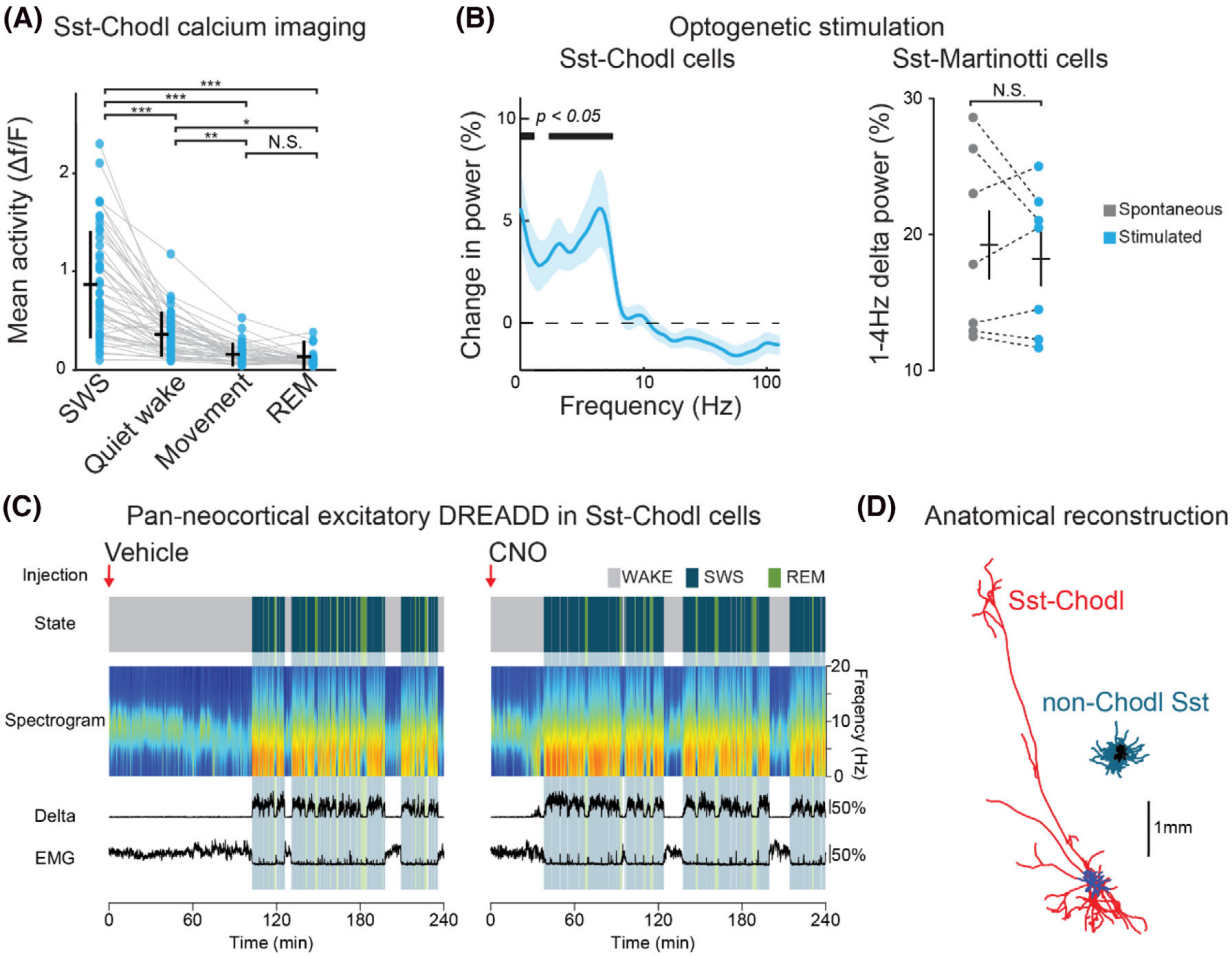
Cortical control of sleep through long‐range inhibition. (A) Mean Sst‐Chodl cell activity across behavioural states, measured using two‐photon calcium imaging in the primary visual cortex of mice. Sst‐Chodl cells were most active during slow‐wave sleep (SWS) and quiet wakefulness (quiet wake) and suppressed during movement and rapid eye movement (REM) sleep. (B) Optogenetic strategies for activating Sst‐Chodl cells (left) and Martinotti cells, another class of *Sst*‐expressing interneurons (right). Activation of Sst‐Chodl cells robustly increased low‐frequency power, particularly in the delta range (1‒4Hz), whereas the same stimulation of Martinotti cells did not alter delta power. (C) Example recordings from a mouse during the dark (active) phase following administration of vehicle (left) or the DREADD agonist clozapine‐N‐oxide (CNO; 0.5 mg kg^−^^1^; right), showing sleep–wake scoring and associated physiological measurements. The mouse expressed excitatory DREADDs in Sst‐Chodl cells throughout the neocortex, and activation of these cells increased sleep duration and shortened sleep latency. (D) Single‐cell reconstructions of an Sst‐Chodl cell and another Sst cell lacking *Chodl* expression (non‐Chodl Sst). Axons are shown in red and blue and dendrites in dark blue and black, respectively. This comparison highlights that Sst‐Chodl cells, unlike most cortical inhibitory neurons (INs), possess long‐range projections spanning millimeters. Figure panels adapted from ref. under CC BY 4.0.

Several important questions remain. We do not yet know how loss of function of Sst‐Chodl neuron function impacts sleep, what drives their recruitment as sleep pressure builds, or how they interact with canonical subcortical sleep‐promoting systems. Future work will also be important to determine whether different cortical regions use these neurons similarly and whether their long‐range organisation contributes to the spatial propagation and coordination of sleep‐related activity.

Sleep disruption is common across neurological, neurodevelopmental and psychiatric disorders, and altered cortical inhibition is a feature of several of these conditions.^8^ In a mouse model of 15q13.3 microdeletion syndrome, Sst‐Chodl neurons showed the largest gene‐expression alterations among neuronal subtypes, and chemogenetic inhibition of their abnormally elevated activity rescued the associated sleep disturbances.^9^ Molecular alterations in Sst‐Chodl neurons have also been reported in the prefrontal cortex of individuals with schizophrenia^10^ providing evidence that this neuronal population may be affected in human brain disorders. Although our experiments were performed in mice, these emerging links to psychiatric and neurodevelopmental conditions provide a rationale for investigating whether the sleep‐regulatory functions of Sst‐Chodl neurons similarly contribute to sleep regulation in humans and whether their dysfunction contributes to associated sleep disturbances. The present findings therefore do not identify an immediate therapeutic target, and the translational implications are yet to be established. However, our study reveals a previously underappreciated level of sleep regulation and provides a genetically defined circuit through which its relevance to human physiology and disease can now be investigated.

Understanding sleep will ultimately require explaining not only how the brain switches between behavioural states, but how those states are constructed across distributed neural networks. The identification of a long‐range inhibitory cortical system capable of coordinating cortical activity and promoting sleep suggests that the cortex is not simply put to sleep. In part, it may help put itself to sleep.

## AUTHOR CONTRIBUTIONS

Both authors contributed to the conception and writing of the manuscript and reviewed and approved the final manuscript.

## CONFLICT OF INTEREST STATEMENT

The authors declare they have no conflict of interest.

## ETHICS STATEMENT

Not applicable.

## Data Availability

No new data were generated or analysed for this article. All the data presented in the figure were adapted from ref. 5 under CC BY 4.0, https://creativecommons.org/licenses/by/4.0/.
